# An Automated Surveillance System (SurCeGGID) for the French Sexually Transmitted Infection Clinics: Epidemiological Monitoring Study

**DOI:** 10.2196/31136

**Published:** 2022-10-25

**Authors:** Ndeindo Ndeikoundam Ngangro, Corinne Pioche, Sophie Vaux, Delphine Viriot, Julien Durand, Bénédicte Berat, Mohammed Hamdaoui, Florence Lot

**Affiliations:** 1 Infectious Diseases Department Sante publique France Saint-Maurice France

**Keywords:** HIV, hepatitis B, hepatitis C, STI, sexual health, epidemiology, surveillance

## Abstract

**Background:**

Viral and bacterial sexually transmitted infections (STIs) are public health concerns worldwide, but surveillance systems are not comprehensive enough to design and monitor accurately STI control strategies in most countries. In 2016, 320 STI clinics (CeGIDDs in French) were implemented in France, primarily targeting most exposed populations, although access is free of charge for anybody.

**Objective:**

This article describes the mandatory surveillance system (SurCeGIDD) based on CeGIDD’s individual data aiming to better guide STI prevention.

**Methods:**

A decree ensured the use of software to manage consultations in CeGIDDs and to transfer surveillance data. A web service was implemented to secure data transfer from CeGIDDs’ software to a centralized database. CeGIDDs can also transfer data in CSV format via a secured data-sharing platform. Then, data are automatically checked before integration. Data on sociodemographic variables, sexual exposure, blood exposure, symptoms, STI tests, STI diagnoses, and sexual health services delivery were collected for the previous year (n–1). Preliminary and descriptive analyses of 2017-2018 data transmitted in 2018 and 2019, respectively, were performed using numbers and proportions for qualitative variables.

**Results:**

In 2017, 54/320 (16.9%) CeGIDDs transmitted their data. In 2018, this number of participants increased to 143/320 (44.7%) CeGIDDs. The corresponding volume of records increased from 2414 in 2017 to 382,890 in 2018. In 2018, most attendances were hospital based (263,480/382,890, 68.81%). In 2018, attendees were mostly men 227,326/379,921 (59.84%), while 151,963/379,921 (40%) were women 632/379,921 (0.17%) transgenders. The median age was 27 years for men, 23 years for women, and 30 years for transgender. Half of the attendees (81,964/174,932, 46.85%) were heterosexual men, 69,016/174,932 (39.45%) heterosexual women, 20,764/174,932 (11.87%) men who have sex with men, and 3188/174,932 (1.82%) women who have sex with women. A majority of them were born in France (227,698/286,289, 79.53%) and unemployed 115,913/211,707 (54.75%). The positivity rates were 0.37% for 205,348 HIV serologies, 1.31% for 131,551 hepatitis B virus serologies, 7.16% for 161,241 *Chlamydia trachomatis* PCR, 2.83% for 146,649 gonorrhea PCR, 1.04% for the syphilis combination of treponema and nontreponema serologies, and 5.96% for 13,313 *Mycoplasma genitalium* PCR.

**Conclusions:**

Despite challenges, the effectiveness of the SurCeGIDD surveillance based on routine patients’ records was demonstrated. The wide range of information, including socioeconomic determinants, might help to better guide and evaluate the prevention policies and services delivery. However, the growing volumes of information will require adapted tools and algorithms for the data management and analyses.

## Introduction

Viral and bacterial sexually transmitted infections (STIs) are public health concerns worldwide because of their high frequency, transmissibility, morbidity, potential complications (infertility, pelvic inflammatory diseases, cancers due to the human papillomavirus [HPV], acquired immunodeficiency due to the HIV), attributable mortality, the multidrug-resistance threat, the risk of congenital transmission, and the impact on the sexual health in the absence of an effective treatment or prevention [1-15].

In 2020, 37.7 million people were infected with HIV and 680,000 deaths were attributable to this infection globally [16,17]. There were 570,000 new cervical cancer cases and about 311,000 deaths attributable to the HPV in 2020 [16,18]. In 2019, 296 million people were infected with chronic hepatitis B, with 1.5 million newly acquired infections and 500,000 liver cancer cases associated with hepatitis B virus (HBV) and hepatitis C virus (HCV) [16,18]. The number of new cases of STI was estimated at 374 million per year and more than 1 million per day [16]. In 2020, 2.3 million deaths related to HIV, hepatitis, and STI worldwide have been reported [16].

In response, the public health strategies aiming to reach the 2030 Sustainable Development Goals (SDG) by eliminating or controlling these epidemics rely on evidence-based interventions [16]. Therefore, the most relevant epidemiological information is required to guide, implement, and evaluate these responses, monitoring progress toward the SDG. Nevertheless, the representativeness of surveillance data remains a major concern in most countries, limiting their use to draw evidence-based control strategies at national and subnational levels [12].

In France, the national sexual health strategy was launched in 2017, aiming to protect sexual health, and to reduce significantly STI incidence by 2030 [19]. In 2016, 320 new STI clinics (Centres gratuits d’information, de dépistage et de diagnostic [CeGIDDs]) were implemented across the country to replace the centers for HIV and viral hepatitis testing and the former STI clinics that were exclusively dedicated to the bacterial STI testing [8,20]. Compared with the former clinics, a wider range of sexual health services were implemented, including STI prevention, testing and treatment, consultations for sexual dysfunctions, sexual violence, sexual education, contraception, and reference to other specialists (eg, proctologist, oncologist) [8,20,21]. Although the CeGIDDs primarily target the most vulnerable populations (men who have sex with men, migrants from endemic countries), the delivery of services is free of charge for the whole population [8,20,21].

Before the implementation of the CeGIDDs, the surveillance systems were based on the sentinel networks for bacterial STI and the mandatory notification for HIV and HBV infections [22-24]. However, their representativeness, sensitivity, timeliness, and flexibility remained challenges even after several decades [22-26]. Indeed, these surveillance systems provided a reliable information at the national level but these indicators could neither describe accurately the epidemics at a lower level, nor give a relevant insight about the CeGIDD users by subregions [12,27].

Since 2016, the CeGIDDs submit a mandatory activity report to the regional health authority each year [16]. As the aggregated data of this report are insufficient to guide the policies [8], the decree of July 2015 ensures that each CeGIDD uses a software “to manage the patients’ records and facilitate the mandatory transmission of case-based data” to Santé publique France (SpF) [21], to establish a comprehensive surveillance of the CeGIDDs.

This article describes SurCeGIDD, the French automated surveillance system of several pathologies, designed to collect case-based data from a wide range of sexual health services, to better guide prevention policies at the national and subnational levels.

## Methods

### Principles of the SurCeGIDD Surveillance

**Figure 1:**
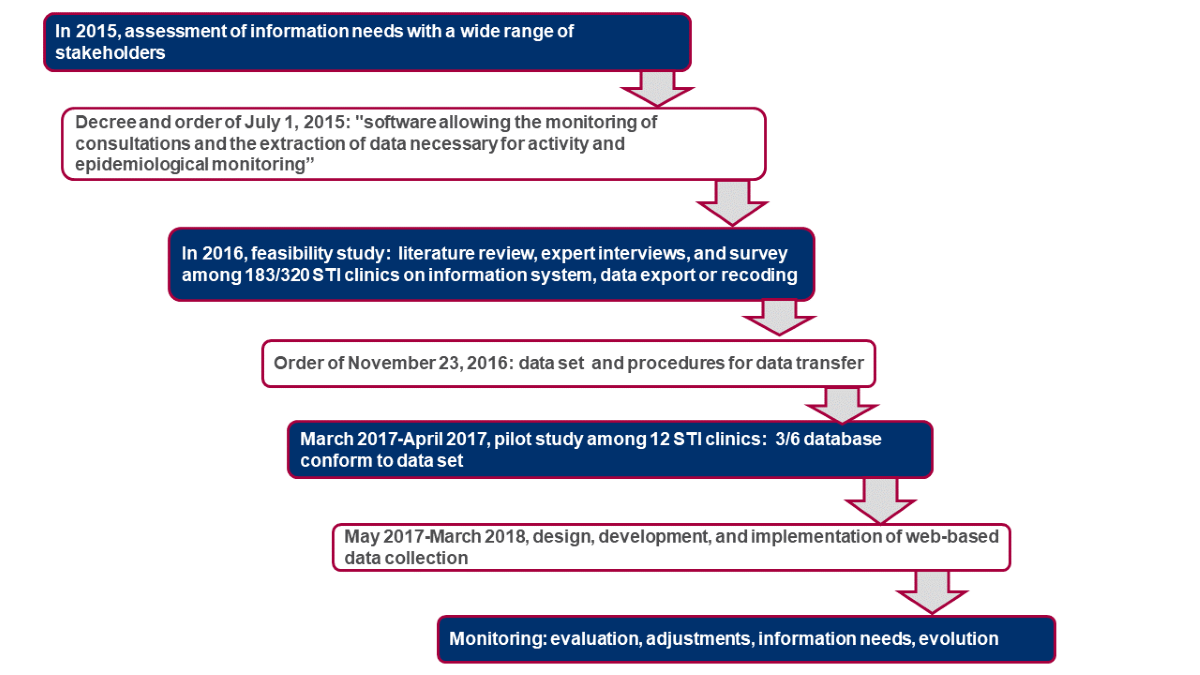
Iterative stages of the SurCeGIDD surveillance project. STI: sexually transmitted infection.

The SurCeGIDD surveillance was developed through a collaborative and iterative approach launched in 2015 (Figure 1). First, the stakeholders engaged in STI clinics, infectious diseases, sexual health promotion, epidemiological surveillance, regional and national health administration, and population representatives were identified through a national concertation meeting held by the French Ministry of Health. The software companies were not engaged in the working groups to prevent any conflict of interest, assuming that the CeGIDDs will contract the implementation of the specifications for SurCeGIDD afterward.

Among the stakeholders, volunteers—regional health agencies (n=3), former STI clinics and HIV-hepatitis testing centers (n=4), charities (n=2), regional response coordination consortium for HIV infection (n=1), SpF, the Health Care and Health Directorates of the Ministry of Health—defined the core principles for the SurCeGIDD surveillance:

- to produce the most relevant epidemiological indicators to guide, implement, and monitor the national sexual health strategy at national, subnational, and local levels;
- to better understand the drivers of these epidemics in France;
- to cover STIs that are not targeted by the classical surveillance in France (genital warts, herpes, *Mycoplasma genitalium*);
- to collect the tests and diagnoses to monitor the positivity rates of STI;
- to cover all sexual health services provided by the CeGIDDs (eg, contraception, HIV preexposure prophylaxis [PrEP], HIV postexposure prophylaxis, vaccination);
- to use patients’ records routinely collected for consultation purposes;
- to use computer-based protocols to facilitate a timely transmission of the required data (no paper form, no online form) and to maximize the coverage of the surveillance;
- to conform to the SurCeGIDD metadata set to enable the comparisons of CeGIDDs, subnational regions, and exposed populations;
- to guarantee patients’ confidentiality by collecting and sharing anonymous data; and
- to secure the data transfer and repository.

Subsequently, the stakeholders mapped the information needs at the national, subnational, and local levels (Textbox 1 [28]). Finally, SpF used this mapping exercise to propose a detailed metadata set that was discussed, amended, and agreed upon by all the stakeholders.

**Variables collected by the SurCeGIDD surveillance, France from the specification for the surveillance of STI clinics.**

**Mandatory Variables**

*Unique identifier*: Clinic, patient, and consultation.

*Sociodemographic variable*: Sex, age, country of birth, date of arrival for migrants, region and department of residence, health insurance coverage.

*Context of consultation*: Anonymity of consultations, outreach testing, use of rapid diagnosis test (point of care).

*History of sexually transmitted infection(s)*: Diagnosis of HIV, hepatitis B virus (HBV), hepatitis C virus (HCV), bacterial sexually transmitted infection (STI).

*Sexual exposure*: Sex of partner(s), number of sexual partners in the last 12 months, condom use by sexual practice with steady or casual partner(s) or both.

*Blood exposure*: Injection drug use, health care in a foreign country, blood transfusion before 1992, contact with person(s) infected with HBV, HCV, or HIV.

*Symptoms*: Presence/absence of symptoms (HIV or STI).

*Tests, diagnoses*: Tests for HIV, HBV, HCV, syphilis, *Neisseria gonorrhoeae*, lymphogranuloma venereum, *Mycoplasma genitalium*. Diagnoses of HIV, HBV, HCV, syphilis, *N. gonorrhoeae*, *Chlamydia trachomatis*, *Mycoplasma genitalium*, genital warts, herpes, antimicrobial resistance of *N. gonorrhoeae*.

*Prescription:* Treatments, preexposure prophylaxis, postexposition treatment for HIV, and vaccination (HBV, hepatitis A virus, human papillomavirus).

**Optional Variables**

*Sociodemographic variable*: Employment status, occupation.

*Context of consultation and history of STI*: Reason for initial consultation in this STI clinic, date of last HIV test, diagnosis of STI in the last 12 months.

*Sexual exposures*: Sex for money, service, drug, house.

*Sexual health services*: Emergency contraception, standard contraception, patient transfer inside the clinic or outside for HIV treatment, HBV and HCV treatment, gynecology, obstetrics, proctology, welfare/social service, sexology, postexposition treatment, preexposure prophylaxis, vaccination, other services.

### Feasibility Assessment

Second, to assess the applicability of the SurCeGIDD principles, SpF conducted a nonsystematic review of scientific articles and the gray literature using the keywords (surveillance or individual or cases-based data extraction or data export or data workflow or data flow or interoperability and France or Europe or the names of comparable industrialized countries [eg, United States of America, United Kingdom, England, Germany]) on PubMed, Embase, Scopus, and Google Scholar. The websites of the national public health agencies, references of interesting articles, and the documents shared by the European networks for surveillances were also checked. After scanning the titles and abstracts for the most relevant ones, articles were analyzed using a form to gather information of interest (eg, framework, metadata set, stakeholder, data collection, data sharing, data integration). The use of health care reimbursement data (no clinical details), the mandatory notification system for HIV (electronic or paper form, such as e-DO in France), and the syndromic surveillance system based on algorithms analyzing the global activity of health care services (no biological details) were excluded from this document review, considering the principle of using clinical and biological records routinely collected during the consultations [23,29-32].

In France, the literature review shows that the surveillance of the colorectal cancer relies on a validated data set of clinical and biological information [33]. This surveillance covers 50 screening centers using 3 software to manage the patients’ records. In the absence of interoperability, an application was developed to recode, extract, and transfer anonymized data from the screening centers to SpF through a secured data-sharing platform [33]. An intermittent data workflow is generated yearly by the screening centers, by activating a specific command on their computer [33]. Then, incoming data are checked and merged using SAS (SAS Institute Inc.) [33]. Another approach is an automated data extraction from 3 multisite laboratories (3Labos surveillance) including tests and diagnoses for 11 pathogens [34]. The 3Labos surveillance relies on a daily/monthly transmission (depending on pathogens) of individual data (sex, age, residence, test, and diagnosis) to SpF through a web service (a protocol enabling the communication between 2 electronic devices via internet) [34]. The incoming data are checked and merged using a Stata (StataCorp) script [34].

The literature review also showed the advantages and limitations of surveillance systems based on data extraction in other countries as well as the development stages of these surveillance systems: GUMCAD (the Genitourinary Medicine Clinic Activity Dataset) in England; CIDR (Computerised Infectious Disease Reporting) in Ireland; SSuN (STD Surveillance Network) in the United States; MIBA (the Danish Microbiology Database) in Denmark; ACCESS (Australian Collaboration for Coordinated Enhanced Sentinel Surveillance) in Australia; eCR (Electronic Case Reporting) in Utah, USA; SOAP (Dutch STI Database) in the Netherlands; and MAVEN (Massachusetts Virtual Epidemiologic Network) in the United States [35-44]. The main points in the development of these electronic and automated surveillance systems were the precise data specifications, the stakeholder’s involvement, the effective information systems to manage patients’ records, and the development and the maintenance of an information platform by the surveillance programs to collect and integrate the data [35-39,44,45]. A long-term strategic framework for the sustainability and the evolution of the surveillance system is also an asset.

Third, to better interpret the information retrieved from the literature, SpF conducted a series of in-depth interviews with French experts (clinicians, biologists, software specialists, data managers, and epidemiologists), using an interview guide to complete the information on the French context. Then, SpF organized a concertation meeting with several clinicians, biologists, and public health specialists to share the findings of the literature review and the interviews, and to discuss the informatics capabilities of the CeGIDDs as well as the levers and obstacles that could impact the implementation of the SurCeGIDD principles in each subnational region.

Fourth, in May 2016, SpF conducted a feasibility survey, by sending an electronic questionnaire to all CeGIDDs. This questionnaire was used to collect information on the computerization of the CeGIDDs, the specifications of their software for the management of the patients records, and their capability to export or recode and transfer a data set in a predefined CSV format. The response rate was 57.2% (183/320). Among the respondents, most CeGIDDs (147/183, 80.3%) used informatic tools to manage their patients/users. A majority of them could export individual data (117/183, 63.9%). Less than half of the CeGIDDs (79/183, 43.2%) were capable of recoding a data set but only 21.9% (40/183) reported being able to recode and transfer data according to a template. The CeGIDDs used mainly 6 software and numerous “homemade” applications. Interestingly, 2 major software companies decided to apply the SurCeGIDD principles at this stage, and communicated their decision to use the surveillance specifications as soon as they would be published. Few months after the feasibility study, one of these companies covered half of the CeGIDDs.

### Surveillance Architecture

Fifth, the findings of the feasibility assessment were used to adjust the SurCeGIDD metadata set, taking into account the day-to-day practices in the CeGIDDs and their informatic limitation. Then, the order of November 23, 2016, stated the mandatory format for the SurCeGIDD data as well as the procedures for data transfer [46]. The specifications for the surveillance were subsequently published on the SpF website [46].

**Figure 2:**
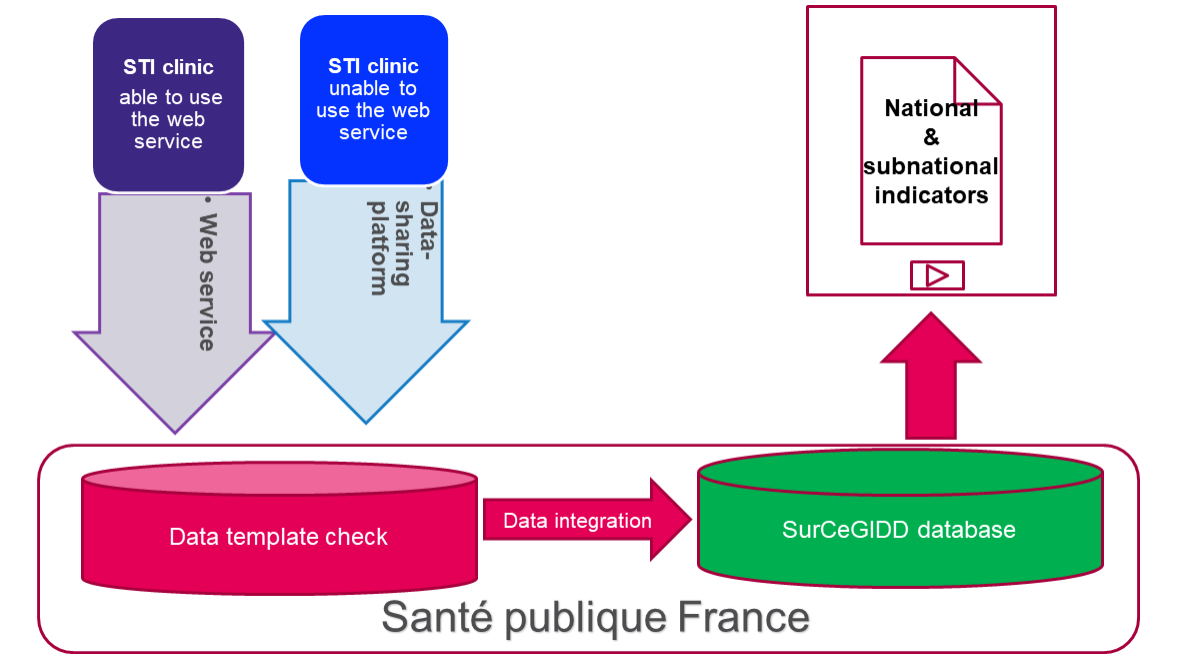
Workflow of sexual health and sexually transmitted infection (STI) data, SurCeGIDD surveillance, France.

Considering the SurCeGIDD specifications, the literature review, and the results of the feasibility survey, a web service was designed for the data transmission [42,47]. If any software provider developed an access to the web service using the specifications shared by SpF, the CeGIDD can automatically transfer its individual database to SpF (Figure 2). Otherwise, the CeGIDDs can export and recode their data in the expected CSV format before their transmission to SpF via a secured data-sharing platform.

Then, the structure of the data is automatically checked and validated before its integration in the SurCeGIDD repository or its return to the CeGIDDs if this control stage fails. During this stage, empty variables are accepted. Warning messages pop-up if the data specifications are not applied. In such a case, SpF helps the CeGIDDs to correct its data set.

A pilot study conducted by SpF among 12 voluntary CeGIDDs helped to test, correct, redesign, and implement the surveillance system between March and April 2017. During the pilot development, 6 CeGIDDs failed to extract their data. Among the 6 data sets that were transmitted to SpF, only 3 were extracted and transferred according to the SurCeGIDD specifications. This highlighted CeGIDDs’ difficulties in performing effectively the expected data management, and the need for friendly software requiring no manual tasks to prepare the data.

### Preliminary Statistical Analyses

Preliminary and descriptive analyses of 2017-2018 data were performed using numbers and proportions to assess the data quality and estimate for the first time the positivity rates for STI in the CeGIDDs (number of positive tests divided by the total of tests), giving an overview of the SurCeGIDD surveillance system after its deployment. Stata 14.2 was used for statistical analyses.

### Ethical Consideration

The protocol was reviewed and approved by the data protection officer of SpF. Then, ethical approval was granted by the French Personal Data Protection Authority (CNIL authorization no. 2049450). Data are collected with patients’ consent. Only anonymized data are transmitted through the SurCeGIDD system. Subsequently, any potential identifying information is aggregated or suppressed after the elimination of duplicates to ensure patients confidentiality. Access to database is strictly restricted to the authorized agents of SpF.

## Results

The number of CeGIDD respondents increased from 54/320 (16.9%) in 2017 to 143/320 (44.7%) in 2018. In 2018, 65.7% (94/143) of respondent CeGIDDs transmitted their data using the web service versus 41% (22/54) in 2017. The volume of corresponding records increased from 2414 in 2017 to 382,890 in 2018.

Assessment of the 2018 data quality showed a high level of completeness for some variables: 93%-99% for sex (382,733/382,890, 99.96%), age (366,501/382,890, 95.72%), HIV (378,499/382,890, 98.85%), HBV (370,562/382,890, 96.78%), HCV (365,591/382,890, 95.48%), syphilis (372,002/382,890, 97.16%), chlamydia (368,930/382,890, 96.35%), and gonorrhea tests (367,622/382,890, 6.01%). Nevertheless, the proportion of missing information was greater than 50% for the number and sex of sexual partners in last 12 months (311,703/382,890, 81.41%), PrEP prescription (296,399/382,890, 77.41%), HBV vaccine (325,215/382,890, 84.94%), hepatitis A virus (HAV) vaccine (347,429/382,890, 90.74%), HPV vaccine (338,165/382,890, 88.32%), diagnoses of genital warts (269,492/382,890, 70.38%), diagnoses of herpes (269,489/382,890, 70.38%), and antimicrobial resistance of *Neisseria gonorrhoeae*. Most respondent CeGIDDs reported ongoing efforts to complete next data sets. Numerous CeGIDDs reported different identifiers for the consultations of the same person, whereas others attributed a unique identifier to the same person regardless of the number of his/her visits. Consequently, it was not possible to link repeated consultations of the same person in SurCeGIDD. An advisory committee for SurCeGIDD was set up to better guide the construction of algorithms enabling effective data management and analyses. Accurate analyses will be performed afterward.

Nevertheless, the first analyses showed that attendees were mostly men 227,326/379,921 (59.84%), whereas 151,963/379,921 (40%) were women and 632/379,921 (0.17%) transgenders in 2018. The median age was 27 years for men, 23 years for women, and 30 years for transgender. Half of the attendees (81,964/174,932, 46.85%) were heterosexual men, 69,016/174,932 (39.45%) heterosexual women, 20,764/174,932 (11.87%) men who have sex with men, and 3188/174,932 (1.82%) women who have sex with women. A majority of them were born in France (227,698/286,289, 79.53%) and unemployed (115,913/211,707, 54.75%). The positivity rates were 0.37% for 205,348 HIV serologies, 1.31% for 131,551 HBV serologies, 7.16% for 161,241 *Chlamydia trachomatis* PCR, 2.83% for 146,649 gonorrhea PCR, 1.04% for the syphilis combination of treponema and nontreponema serologies, and 5.96% for 13,313 *M. genitalium* PCR.

## Discussion

### Principal Findings

Despite numerous challenges, SurCeGIDD surveillance was successfully implemented. The effectiveness of this system to collect automatically important volumes of data was demonstrated by 143/320 (44.7%) CeGIDDs that reported simultaneously the STI tests, diagnoses, clinical details, and patients’ socioeconomic characteristics for the first time in France. Although these data need further consolidation, the preliminary analysis confirmed that CeGIDDs’ attendees are particularly exposed to STI with high positivity rates: 0.37% for HIV, 1.31% for HBV, 7.16% for Ct, 2.83% for gonorrhea, 1.04% for syphilis, and 5.96% for *M. genitalium.* These results also gave the first insight into *M. genitalium* testing in the CeGIDDs. Further analyses will better describe their situation.

However, a selection of CeGIDDs with greater human, informatic, or financial resources compared with the nonparticipating structures cannot be ruled out. But the participation rate of 143/320 (44.7%) will probably increase in the next years, considering the growing interest of the CeGIDDs and software providers for SurCeGIDD data. Experiences in STI surveillance in Denmark, England, Ireland, Australia, and United Sates seem to confirm this expectation [35-38,44]. Moreover, the increasing computerization of the CeGIDDs and the adapted support provided by SpF in the use of the specifications might contribute to a better representativeness of the data in the medium term.

This support is also an opportunity for SpF to correct the metadata set, taking into account the operational feedback from the structures. Nevertheless, the time to develop or modify the informatic platforms was underestimated by SpF and stakeholders, delaying the use of the updated metadata set in data collection. This was also observed in other contexts, and it must be better anticipated in informatic development [35-38,44]. Moreover, difficulties to extract or recode data by CeGIDDs according to the specifications hampered the surveillance implementation. Indeed, some CeGIDDs selected a range of variables considering feasibility burden. Consequently, nonprioritized variables were mostly missing.

The proportion of missing information was also greater than 50% for important variables such as the characteristics of sexual partners (311,703/382,890, 81.41%), PrEP use (296,399/382,890, 77.41%), HBV vaccine (325,215/382,890, 84.94%), HAV (347,429/382,890, 90.74%), HPV vaccine (338,165/382,890, 88.32%), diagnoses of genital warts (269,492/382,890, 70.38%), diagnoses of herpes (269,489/382,890, 70.38%), and antimicrobial resistance of *N. gonorrhoeae.* Some patient’s records could not be recoded despite the CeGIDDs’ willingness to conform to the surveillance specifications. For example, some CeGIDDs only recorded trademarks of vaccines instead of the names and doses that enable the monitoring of vaccination coverage. Some adjustment might be necessary in the data format to better take CeGIDDs’ needs into account. This also underlines the need for appropriate human and financial resources to support CeGIDDs’ adherence to the specifications. Nevertheless, data transmission will require no extra labor burden once the specifications have been implemented, in contrast to the use of paper or electronic forms [35,41,42].

Because of the legal context in France, it was not possible to engage software companies in the SurCeGIDD working groups. In England, software providers contributed to the early stage of the surveillance project, applying GUMCAD specification in the development of their tools [36]. Moreover, the lack of specific funding for information systems in CeGIDDs might limit SurCeGIDD’s expansion and evolution because each structure has to pay for the implementation of the specification changes. Conversely, incentives and additional funding contributed to ensure the largest and long-term coverage in other contexts [35,37,41,42]. Mandatory use of national specifications by all software companies might be a solution. It might not only enable database transmission by all CeGIDDs, but can also facilitate the implementation of any change simultaneously in all CeGIDDs (additional information) [35,36].

Regarding SurCeGIDD coverage, the list of CeGIDDs including emails and telephone numbers was not updated since 2016, despite the creation of new structures and closures of other ones by regional health agencies [8]. Therefore, it was not possible to engage with noninformed CeGIDDs in the surveillance. SpF advocates for a systematic transmission of updated phone books by regional public health agencies each year, to ensure the best representativeness of the surveillance data.

Considering the volumes of data shared, SpF needs to upgrade its tools and procedures, using algorithms to automatize data monitoring, quality checking, and epidemiological reports at national and subnational levels. There is also a need to formalize rules and process for data sharing and analyses [35,48,49].

Despite its limitations, SurCeGIDD surveillance creates new opportunities for research collaboration on sexual health in France [35,41,50]. Availability of a wide range of individual characteristics (health insurance, occupation) in this database will be an opportunity to better understand the impact of social health inequalities on STI epidemics in France. SurCeGIDD will also help to fill the gap in our knowledge of *M. genitalium*, genital warts, herpes, contraception, and vaccination in France [8]. Generating a continuous data workflow instead of a yearly transmission of data might be the next improvement of SurCeGIDD, to timely detect and investigate STI events (multidrug resistance, uncommon anatomic location, upsurge, clusters) [41,44,51-53]. An anonymous linkage of patients within the SurCeGIDD database might improve the epidemiological surveillance [35,36,42]. The almost real-time transmission of routinely collected data to monitor COVID-19 diagnoses from laboratories in France demonstrated the feasibility of such evolutions [48]. Moreover, the expansion of this laboratory surveillance toward STI diagnoses might help to complete the picture of STI epidemics in France.

### Conclusions

The automated SurCeGIDD surveillance is functional despite the challenges that hamper its coverage expansion. A long-term vision relying on sustained funding, including informatic developments for STI clinics, and the mandatory use of the specifications by software providers might help to improve the repressiveness of surveillance data to better guide and evaluate prevention strategies and services delivery. Indeed, the timeliness and efficacy of the COVID-19 monitoring systems demonstrate how electronic medical records could effectively replace passive surveillance systems, particularly sentinel surveillance systems commonly overwhelmed by important volumes of information. Moreover, exhaustive medical and laboratory records could help to compare more accurately countries for STI trends, drivers of epidemics, and sexual health services delivery.

## Abbreviations

ACCESS: Australian Collaboration for Coordinated Enhanced Sentinel Surveillance
CeGIDD: Centre gratuits d’information, de dépistage et de diagnostic
CIDR: Computerised Infectious Disease Reporting
eCR: Electronic Case Reporting
GUMCAD: Genitourinary Medicine Clinic Activity Dataset
HAV: hepatitis A virus
HBV: hepatitis B virus
HCV: hepatitis C virus
HPV: human papillomavirus
MAVEN: Massachusetts Virtual Epidemiologic Network
MIBA: Danish Microbiology Database
PrEP: preexposure prophylaxis
SDG: Sustainable Development Goals
SOAP: Dutch STI Database
SpF: Santé publique France
SSuN: STD Surveillance Network
STI: sexually transmitted infection
